# Protective Effect of Tetracycline against Dermal Toxicity Induced by Jellyfish Venom

**DOI:** 10.1371/journal.pone.0057658

**Published:** 2013-03-11

**Authors:** Changkeun Kang, Yeung Bae Jin, Jeongsoo Kwak, Hongseok Jung, Won Duk Yoon, Tae-Jin Yoon, Jong-Shu Kim, Euikyung Kim

**Affiliations:** 1 College of Veterinary Medicine, Gyeongsang National University, Jinju, Korea; 2 Headquarters for Marine Environment, National Fisheries Research & Development Institute, Shiran-ri, Gijang-eup, Gijang-gun, Busan, Korea; 3 Department of Dermatology and Institute of Health Sciences, School of Medicine, Gyeongsang National University, Jinju, Korea; 4 Research Institute of Life Science, Gyeongsang National University, Jinju, South Korea; Broad Institute of Harvard and MIT, United States of America

## Abstract

**Background:**

Previously, we have reported that most, if not all, of the Scyphozoan jellyfish venoms contain multiple components of metalloproteinases, which apparently linked to the venom toxicity. Further, it is also well known that there is a positive correlation between the inflammatory reaction of dermal tissues and their tissue metalloproteinase activity. Based on these, the use of metalloproteinase inhibitors appears to be a promising therapeutic alternative for the treatment of jellyfish envenomation.

**Methodology and Principal Findings:**

Tetracycline (a metalloproteinase inhibitor) has been examined for its activity to reduce or prevent the dermal toxicity induced by *Nemopilema nomurai* (Scyphozoa: Rhizostomeae) jellyfish venom (NnV) using *in vitro* and *in vivo* models. HaCaT (human keratinocyte) and NIH3T3 (mouse fibroblast) incubated with NnV showed decreases in cell viability, which is associated with the inductions of metalloproteinase-2 and -9. This result suggests that the use of metalloproteinase inhibitors, such as tetracycline, may prevent the jellyfish venom-mediated local tissue damage. *In vivo* experiments showed that comparing with NnV-alone treatment, tetracycline pre-mixed NnV demonstrated a significantly reduced progression of dermal toxicity upon the inoculation onto rabbit skin.

**Conclusions/Significance:**

It is believed that there has been no previous report on the therapeutic agent of synthetic chemical origin for the treatment of jellyfish venom-induced dermonecrosis based on understanding its mechanism of action except the use of antivenom treatment. Furthermore, the current study, for the first time, has proposed a novel mechanism-based therapeutic intervention for skin damages caused by jellyfish stings.

## Introduction

Over the last decade, unusual large blooms of *N. nomurai* jellyfish have occurred in Yellow sea, East China Sea, and East Sea [1] and the patients stung by this jellyfish species have increased correspondingly. It has been reported that over 2000 cases of *N. nomurai* jellyfish envenomation occurred in the coastal areas of Korea, Japan and China since 1983, including fatal cases in some patients with the jellyfish sting [2]. The venom of *N. nomurai* jellyfish (NnV) contains a variety of bioactive proteins that are cytotoxic, hemolytic, hepatotoxic, and cardiotoxic [3]–[5]. The cutaneous symptoms caused by this jellyfish stings were very painful with a strong burning sensation, followed by erythematous eruption with small vesicles [6]. In a previous report, we have shown that most, if not all, of the Scyphozoan jellyfish venoms contain multiple components of various metalloproteinases, which largely contribute to their cytotoxic activities. All the Scyphozoan jellyfish venoms examined showed gelatinolytic, caseinolytic, and fibrinolytic activities, each of which contains a multitude of enzyme components with molecular weights between 17 and 130 kDa [7]. Based on our findings, it is very likely that these metalloproteinases play some important role in the pathologic processes of jellyfish envenomation.

Meanwhile, the most severe of all jellyfish stings comes from the box jellyfish, *Chironex fleckeri*. Following envenomation by this jellyfish, the major life-threatening events are the systemic manifestations, affecting both heart and lungs. The box jellyfish venom is cardiotoxic, and the lungs can become congested as a secondary effect of the cardiac arrest [8]. Furthermore, there is usually extreme pain, with bright red wheal formation which results in necrosis of the affected skin area [9]. The commercial antivenom has been developed against box jellyfish and used since 1970 with questionable success [10]. Although antivenom therapy is a primary treatment of jellyfish envenomation yet especially for the patients stung by *Chironex fleckeri*, many questions still remain about their effectiveness in the clinical application. Except the antivenom therapy, there have been a variety of treatment protocols suggested for alleviating jellyfish sting-associated dermonecrosis. The most used today for this purpose includes prostaglandin inhibitors and antihistamines with limited success. Since the mechanism of action of jellyfish venom is still poorly understood, the definitive treatment for jellyfish envenomation and its dermonecrosis has not yet been established. Although it is widely known that jellyfish stings can induce severe damage in dermal tissue, it has been rarely studied from the etiological point of view. Interestingly enough, the overexpression of MMPs has been implicated in the pathogenesis of several diseases, from cancer to inflammatory conditions [11], being also involved in dermonecrosis induced by spider venom [12]. Recently, inhibitors of matrix metalloproteinases (MMPs) have been used to prevent secondary infections or inhibit the hemorrhagic activities of snake venoms [13],[14]. Tetracycline is an effective MMP inhibitor with diverse clinical applications which do not depend on its antibiotic effect [15]. Thus, it is strongly suspected that tetracycline has a therapeutic potential for the treatment of jellyfish envenomation.

The aim of this study was to investigate the role of MMPs using both *in vitro* and *in vivo* model in the dermal toxicity of *N. nomurai* jellyfish venom. Furthermore, we also examined the ability of tetracycline to inhibit the clinical sequela of *N. nomurai* jellyfish envenomation.

## Materials and Methods

### Chemicals and Reagents

Dulbecco’s Modified Eagle’s Medium (DMEM), penicillin, streptomycin sulfate, trypsin, dimethyl sulfoxide (DMSO), 3-(4,5-dimethylthiazol-2-yl)-2,5-diphenyltetrazolium bromide (MTT), Alsever’s solution and tetracycline were purchased from Sigma-Aldrich Inc. (St. Louis, MO, USA). Human MMP-2 and MMP-9 were from Santa Cruz Biotechnology (Santa Cruz, CA, USA). All other reagents used were of the purest grade available.

### Jellyfish Collection and Preparation

Mature specimens of *N. nomurai* jellyfish were captured from Korea Strait along the coasts of Tongyoung in September, 2007. The samples themselves as well as their collected sites are not privately-owned or protected in any way. Further, *N. nomurai* jellyfish is not an endangered or protected species, it is rather classified as a harmful organism in the waters of Republic of Korea. The tentacles dissected from the jellyfish were stored in ice and transferred immediately to our laboratory for further preparation. Nematocysts were isolated from the dissected tentacles as described by Bloom *et al*. [16] with a slight modification. In brief, tentacles were gently swirled with the addition of distilled water, then stood still for 1∼2 h to remove debris and sea water. After decanting the supernatant, tentacles settled down at the bottom were mixed with 2×(v/v) distilled water and shaken vigorously for 3 min. The detached nematocysts were separated by filtering tentacle preparation through 4 layers of medical gauze. This was repeated for two more times with additional distilled water to harvest nematocysts from the tentacles. The filtrates were centrifuged (700×*g*) at 4°C for 20 min and the pellets (nematocysts) were lyophilized and stored −20°C until use.

### Venom Extraction and Preparation

Venom was extracted from the freeze-dried nematocysts using the technique described by Carrette and Seymour [17] with a minor modification. In brief, venom was extracted from 50 mg of nematocyst using glass beads (approximately 8000 beads; 0.5 mm in diameter) and 1ml of ice-cold (4°C) phosphate buffered saline (PBS, pH 7.4). These samples were shaken in a mini bead mill at 3000 rpm for 30-s intervals for five times with intermittent cooling on ice. The venom extracts were then transferred to a new Eppendorf tube and centrifuged (22,000× *g*) at 4°C for 30 min. This supernatant was used as NCV for the present study. Protein concentration of the venom was determined by the method of Bradford technique [18] (Bio-Rad, C.A. USA) and the venom was used based on its protein concentration.

### Cell Culture and Cytotoxicity Assay

HaCaT (human keratinocyte) and NIH3T3 (mouse fibroblast) cells were used for assessing the cytotoxicity of the venom. Cells were cultured in Dulbecco’s Modified Eagle’s Medium (DMEM) supplemented with 10% fetal bovine serum (FBS), 100 U/ml penicillin, 100 µg/ml streptomycin at 37°C with 5% CO_2_. For cytotoxicity experiment, cells were seeded in 24-well plates at a density of 10^4^ cells per well and cultured for 24 h. Then, they were treated with various concentrations of *N. nomurai* jellyfish venom for additional 24 h. In inhibitory study, *N. nomurai* jellyfish venom was preincubated with indicated concentrations of tetracycline at 37°C for 1 h before being tested for the residual cytotoxic effects. After the experiments, cytotoxicity was assessed by measuring mitochondrial dehydrogenase activity, using 3-(4, 5-dimethylthiazol-2-yl)-2, 5-diphenyltetrazolium bromide (MTT) assay. Briefly, 100 µl of MTT solution (5 mg/ml) was added to each well and incubated for another 3 h at 37°C. After removing the supernatant, the formazan crystal generated was dissolved by adding 250 µl/well of dimethyl sulfoxide (DMSO) and the absorbance was detected at 540 nm using a spectrophotometric microplate reader (BioTek Instruments, Inc., Winooski, USA).

### Gelatin Zymography

MMP-2 and MMP-9 secretion of HaCaT and NIH3T3 cells into culture medium was determined using gelatin zymography [19]. Briefly, HaCaT and NIH3T3 cells were seeded (1 × 10^5^ cells/well) in 6-well and allowed to grow to confluence for 24 h and maintained in DMEM with 10% FBS. The cells were washed with PBS and incubated in serum-free DMEM for 12 h. The supernatants from HaCaT and NIH3T3 cells, collected after 24 h of incubation with venom (5 µg/ml and 10 µg/ml), in the presence or absence of tetracycline (100 µM). The supernatant was collected and mixed with non-reducing sample buffer, then electrophoresed in 10% polyacrylamide gel containing 0.1% (w/v) gelatin. After the electrophoresis, gel was washed for 30 min twice with 2.5% Triton X-100 and incubated for additional 18 h at 37°C for the enzymatic reaction of MMPs in zymography reaction buffer (200 mM NaCl, 10 mM CaCl_2,_ 50 mM Tris-HCl, pH 7.4). The gel was then stained with Coomassie blue R-250 (0.125% Coomassie blue R-250, 50% methanol, 10% acetic acid) and destained (methanol/acetic acid/water, 40/10/50, v/v/v).

### Electrophoresis and Western Blotting

HaCaT and NIH3T3 cells treated with venom (2.5, 5, 10 µg/ml), in the presence or absence of tetracycline (100 µM) for 24 h were rinsed twice with ice-cold PBS, and treated with 100 µl of lysis buffer (50 mM Tris-HCl, pH 7.4, 1% NP-40, 0.25% sodium deoxycholate, 150 mM EDTA, 1 mM PMSF, 1 mM sodium orthovanadate, 1 mM NaF, 1 mM Na_3_VO_4_, 1 µg/ml aprotinin, 1 µg/ml leupeptin, 1 µg/ml pepstatin). The plate was rocked on ice for 3 min, and the scraped samples were allowed to lyse for additional 30 min on ice with periodic vortexing. Cell debris was removed by centrifugation (22,000×*g*, at 4°C for 30 min) and the resulting supernatants were collected for immunoblot analysis. Protein concentrations in cell lysates were determined using a Bio-Rad protein assay reagent (Bio-Rad, C.A. USA). Cytosolic proteins in cell lysates (30–40 µg) were separated on 12% SDS-polyacrylamide gel, transferred to PVDF membranes (Bio-Rad, C.A. USA), and subsequently subjected to immunoblot analysis using specific primary antibodies. After incubation overnight at 4°C with a primary antibody, the membrane was incubated with horseradish peroxidase-conjugated secondary antibody (Cell Signaling Technology, Beverly, MA) for 1 h at room temperature. The blots were visualized by using the enhanced chemiluminescence method (ECL, Amersham Biosciences, Buckinghamshire, UK) on blue light-sensitive film (Fujifilm Corporation, Tokyo, Japan). In some cases, the blotted membranes were stripped and reprobed using other primary antibodies. Densitometric analysis was performed with a Hewlett-Packard scanner and NIH Image software (Image J).

### Experimental Animals

Adult male New Zealand white rabbits weighing approximately 3 kg were purchased from Central Laboratory Animal Inc. (Korea). The rabbits were kept in a separate HEPA-filtered room with negative pressure and 12/12-h light/dark cycle and were housed individually with water and food provided *ad libitum*. All efforts were made to minimize animal suffering and to reduce the number of animals used. The experimental animals were handled in strict accordance with the recommendations in the Guide for the Care and Use of Laboratory Animals of Ministry for Food, Agriculture, Forestry and Fisheries of Republic of Korea. The protocol was approved by the Committee on the Ethics of Animal Experiments of the Gyeongsang National University (Permit Number: GNU-LA-36).

### Dermal Toxicity Test

*Nemopilema nomurai* jellyfish venom (500 µg) was injected intradermally (200 µl) in the appointed partition of shaved dorsum of rabbits. After 1.5 h of inoculation, injection sites were topically treated with lanolin cream alone, or lanolin cream plus tetracycline (5%, w/v, tetracycline cream) twice a day for 48 h. Negative control and positive control groups were the inoculations with PBS (vehicle) or *N. nomurai* jellyfish venom, respectively, without any topical post-treatment. For pre-mixed venom experiment, the venom was pre-incubated with tetracycline (100 µM, 1∶1, v/v) for 1 h at 37°C, then intradermally injected as previously described. The size of lesion was measured at 1.5, 6, 12, 18, 24, 30, 36, 42 and 48 h following injection. The experiments were repeated six times using three rabbits per group.

### Histological Analysis

Male New Zealand white rabbits were inoculated with 500 µg of NnV alone, tetracycline pre-mixed NnV or PBS. After 1.5 h of inoculation, a typical dermonecrotic lesion was detected in the envenomed animals and at this time the treatment with tetracycline was begun. After 48 h, the animals were sacrificed by terminal exsanguination after being anesthetized with ketamine HCl (40 mg/kg) (Fort Dodge Laboratories, Inc., Fort Dodge, Iowa) and xylazine (10 mg/kg) (Bayer Corporation, Agriculture Division, Shawnee Mission, Kans.). The obtained skin sections were fixed in 10% neutralized formalin solution. After fixing, the tissues were dehydrated and embedded in paraffin. The paraffin blocks were sectioned at 5 µm, and then stained with hematoxylin and eosin (H&E) for observing any histological changes.

### Statistical Analysis

The results are expressed as a mean ± standard deviation (S.D.). A paired Student’s *t-*test was used to assess the significance of differences between two mean values. *P*<0.05 was considered to be statistically significant.

## Results

### Cytotoxicity of N. Nomurai Jellyfish Venom

**Figure 1:**
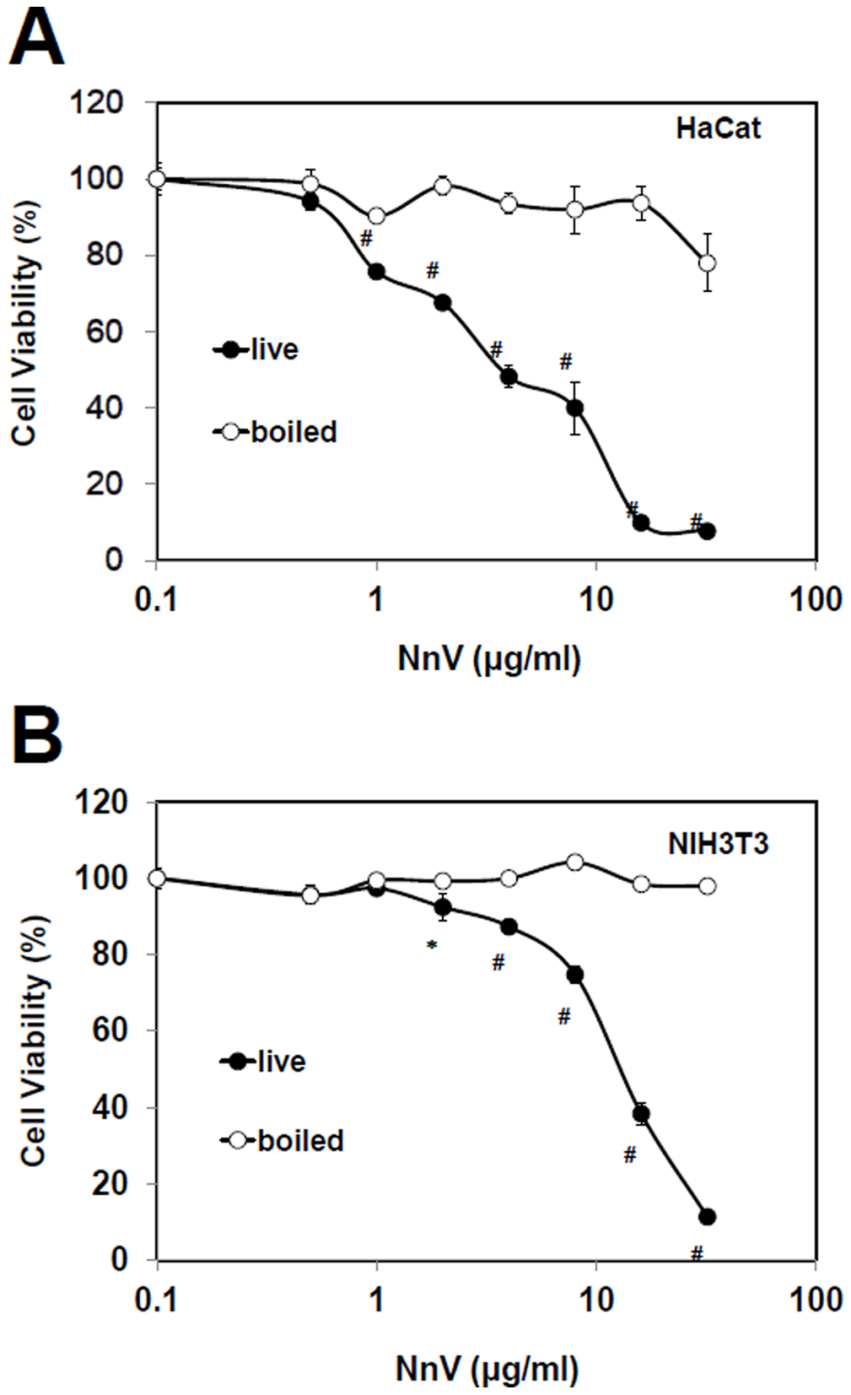
Freshly prepared *N. nomurai* jellyfish venom shows cytotoxic effect on HaCaT (human keratinocyte) and NIH3T3 (mouse fibroblast) cells. Exponentially growing HaCaT (A) and NIH3T3 (B) were treated with various concentration of *N. nomurai* jellyfish venom and with boiling of various concentration of *N. nomurai* jellyfish venom for 24 h. Cells incubated with no venom (PBS) were taken as proper controls. The results are expressed as mean ± S.D. for three independent experiments in triplicates, *P<0.05, ^#^P<0.01, as compared with the control value.

In order to estimate the toxic effects of *N. nomurai* jellyfish venom (NnV), HaCaT and NIH3T3 cells were incubated for 24 h with increasing concentrations of NnV. A comparison of its relative cytotoxicity on the cells was illustrated in Fig. 1. Both HaCaT and NIH3T3 cells showed a venom concentration-dependent cell death and their calculated LC_50_ (the concentration that kills 50% of the cells) were 4.3 and 13.3 µg/ml, respectively. The results reveal that NnV has a cytotoxic component which exhibits much higher potency on HaCaT epithelial cells than NIH3T3 fibroblast cells. The greater susceptibility of epithelial cells to jellyfish venom of the present study appears to have the same connotation as the well-known dermonecrotic pathogenesis of the skin stung by venomous jellyfish species. The venom stability has also been investigated under boiling condition (95°C, 10 min). In this experiment, cytotoxicity of the heat-treated NnV was examined using HaCaT and NIH3T3 cells as described above. Upon the treatment of this boiling condition, the venom drastically lost its cytotoxic activity. This indicates that the toxic component(s) of NnV is highly susceptible to and easily neutralized by the heat treatment.

### Inhibitory Effects of Tetracycline on NnV-induced Cytotoxicity in HaCaT and NIH3T3 Cell

**Figure 2:**
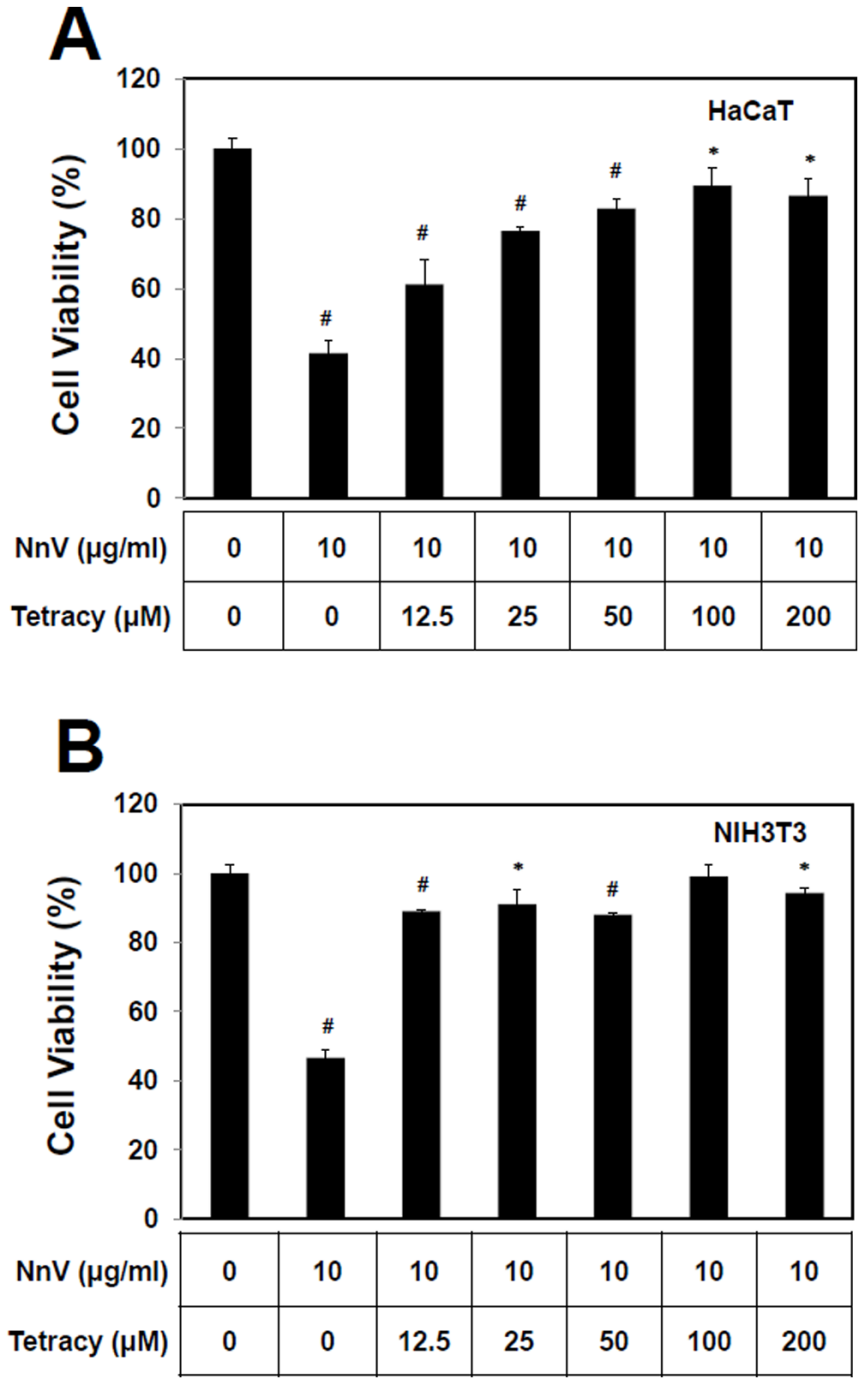
Tetracycline inhibits the cytotoxic effect of *N. nomurai* jellyfish venom on HaCaT (A) and NIH3T3 (B) cells. In brief, the venom aliquots were pre-incubated with each cell of LC50 concentration and various concentrations of tetracycline at 37°C for 1 h. Then, the biological activity of each venom sample was evaluated by assessing its remaining cytotoxic effect after 24 h incubation on HaCaT and NIH3T3 cells as described earlier. The results are expressed as mean ± S.D. for three independent experiments in triplicates, *P<0.05, ^#^P<0.01, as compared with the control value.

To further examine the possible association of cell death and metalloproteinase, HaCaT and NIH3T3 were treated with NnV (LC_50_ of each cell) in the absence or the presence of increasing concentrations of tetracycline. The treatment of tetracycline could fully protect the cells from the cytotoxic effects of NnV at the concentrations of 12.5 µM and 100 µM for NIH3T3 and HaCaT, respectively (Fig. 2). The tetracycline, at the dose of 200 µM, did not affect HaCaT and NIH3T3 cell viability (data not shown).

### Presence of MMPs in HaCaT and NIH3T3 Cells

**Figure 3:**
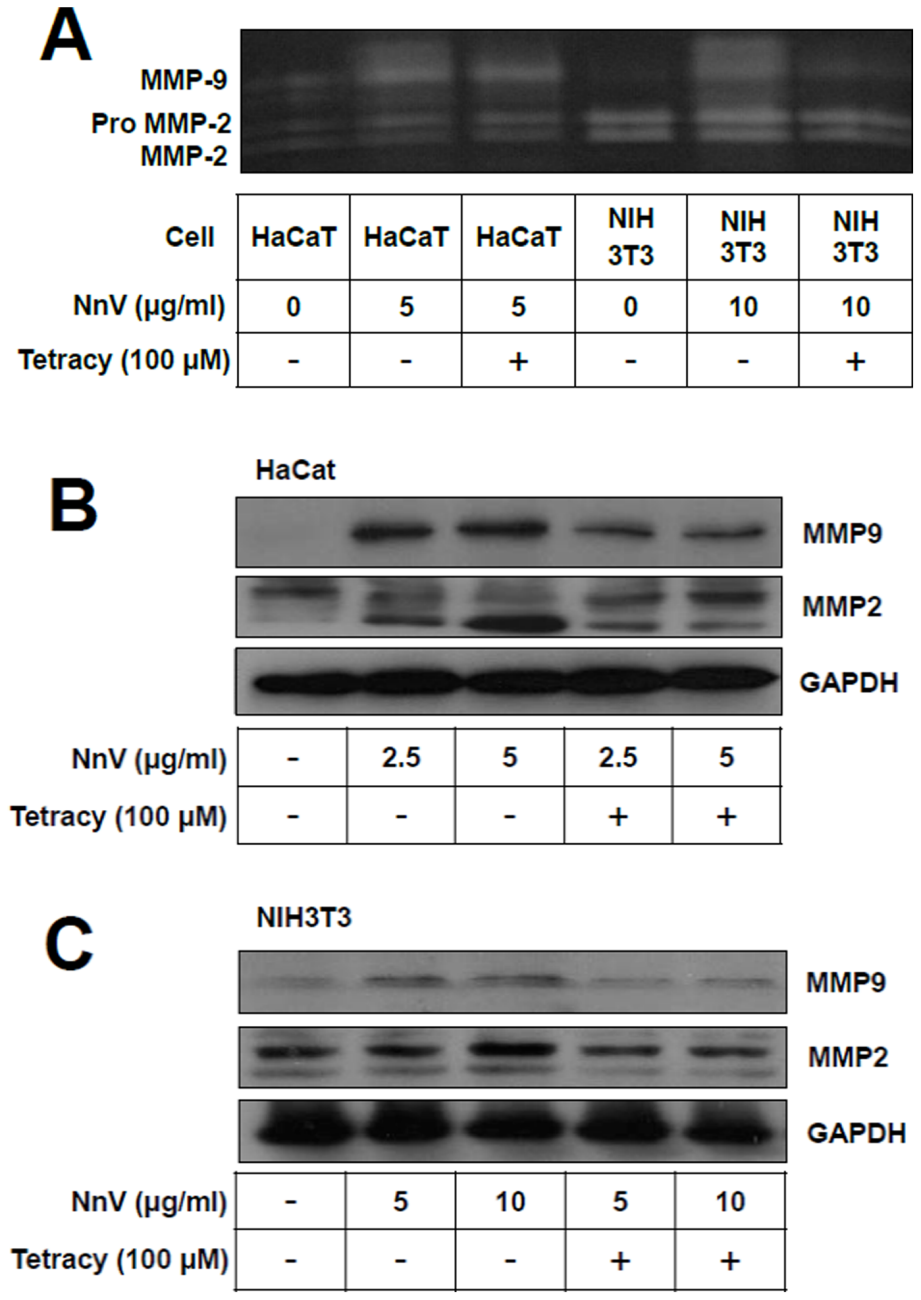
*N. nomurai* jellyfish venom induces the expression of metalloproteinase activities in HaCaT and NIH3T3 cells. The cells were treated for 24 h with indicated concentrations of *N. nomurai* jellyfish venom in the presence or absence of tetracycline (100 µM). Then, the gelatinolytic activities of MMP-2 and MMP-9 in the culture supernatants were detected by electrophoresis on gelatin containing 10% polyacrylamide gel and zymography assay (A). The protein expression level of MMPs in HaCaT (B) and NIH3T3 (C) cells were analyzed by Western blot using the primary antibodies for MMP-2 or MMP-9, respectively. GAPDH protein level was used as a control for equal loading. The results are expressed as mean ± S.D. for three independent experiments in triplicates, *P<0.05, ^#^ *P*<0.01, as compared with the control value.

In order to investigate the possible involvement of MMPs in the jellyfish venom-induced cytotoxicity, HaCaT and NIH3T3 cells were treated with NnV at indicated concentrations (2.5, 5, and/or 10 µg/ml), and then MMPs activities of the cell culture media were analyzed by gelatin zymography and Western blot. Treatment of NnV to the cells significantly enhanced activity-based zymography of both MMP-2 and MMP-9 compared with their respective vehicle treated controls. NnV jellyfish venom-enhanced MMP activities could be suppressed in the presence of tetracycline, a well-known MMP inhibitor (100 µM) (Fig. 3A). Being consistent with this gelatin zymography study, Western blot analyses revealed that NnV treatments stimulate the MMPs expressions, particularly MMP-9. The treatment of tetracycline largely prevented the expression of MMP-2 and MMP-9 activities induced by NnV (Fig 3B and 3C). These results confirmed that NnV can stimulate the secretion/expression of MMP-2 and MMP-9.

### Development of Skin Lesion Induced by N. nomurai Jellyfish Venom

**Figure 4:**
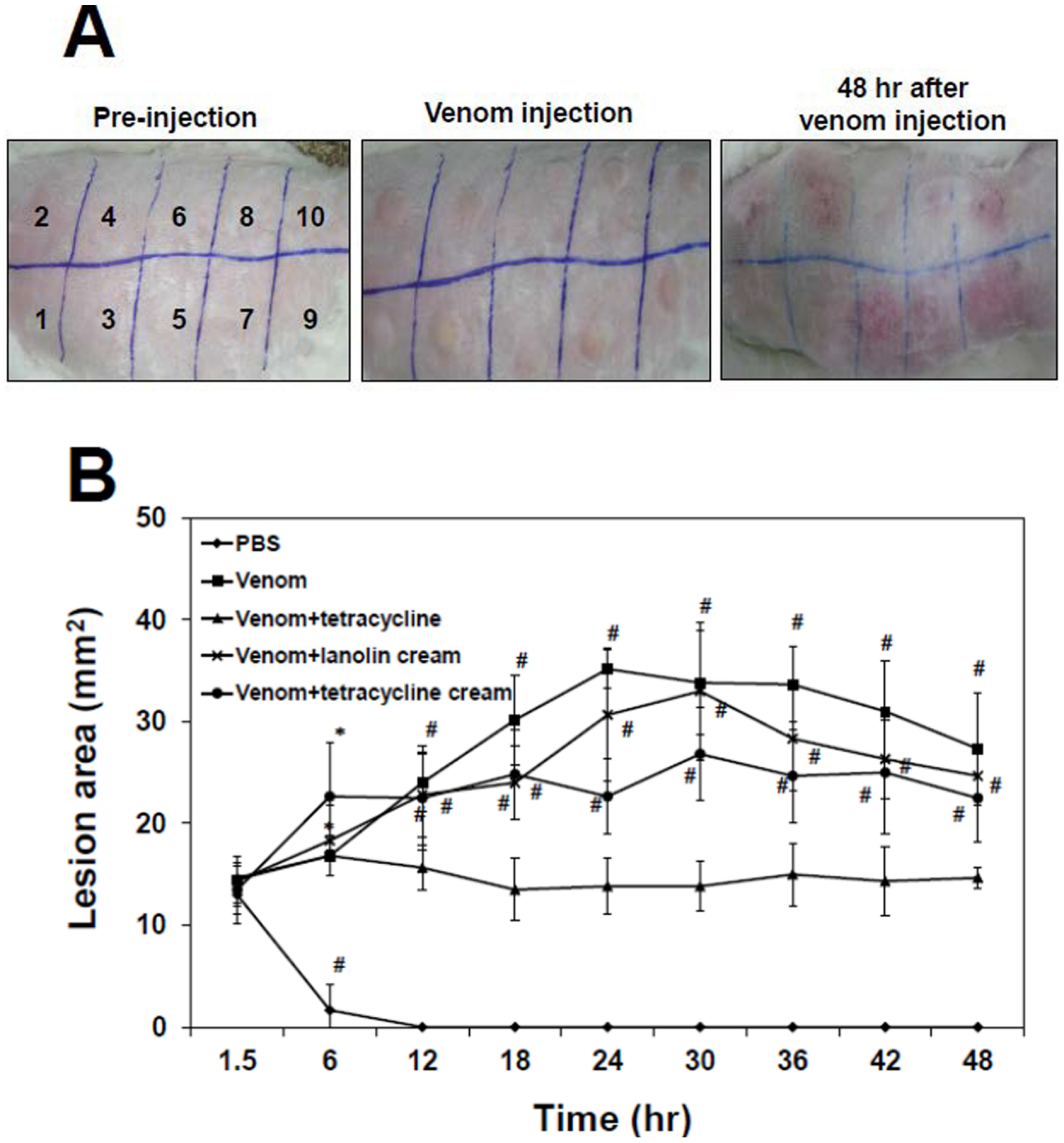
Protective effects of tetracyclines on the development of skin lesion induced by *N. nomurai* jellyfish venom. New Zealand white rabbits were injected intradermally (200 µl) at the numbered sites shown on the dorsum with PBS (**1, 6**); *N. nomurai* jellyfish venom (500 µg, **2, 7**); pre-mixed venom (venom plus tetracycline, preincubated at 37°C for 1 h, **3, 8**); *N. nomurai* jellyfish venom followed by the treatment with lanolin cream (**4, 9**); *N. nomurai* jellyfish venom followed by the treatment with tetracycline cream (**5, 10**), respectively. After 1.5 h of inoculation, a typical dermonecrotic lesion was detected at the sites of venom injection of the experimental animals. From this time point, either lanolin cream alone or tetracycline added lanolin cream had been treated on the venom injection sites and the treatments had been performed twice a day during the next 48 h (A). The numbers of 6∼10 are repeated sites of the numbers of 1∼5, respectively. Time course of the size of skin lesion was also measured at each time points of 1.5, 6, 12, 18, 24, 30, 36, 42, and 48 h of post-inoculation (B). The results are expressed as mean ± S.D. for three independent experiments in twice, *P<0.05, ^#^ *P*<0.01, as compared with the control value.

Adult male New Zealand white rabbits were intradermally inoculated with either phosphate buffered saline (PBS) or 500 µg of NnV, then the size of tissue swelling or skin lesion on the site of injection was measured at 1.5, 6, 12, 18, 24, 30, 36, 42 and 48 h of post-inoculation. The protective effects of tetracycline were examined for the injection of tetracycline-pretreated venom (pretreated with 100 µM tetracycline [1∶1, v/v] for 1 h at 37°C), venom injection plus lanolin cream post-treatment, and venom injection plus tetracycline-added lanolin cream twice a day for 48 h. All of the experimental groups, except PBS control group, produced a well-defined oval area of redness and conspicuous edema (Fig. 4A). Those initial gross changes were most intense after 6 h, but stabilized thereafter, decreasing in color intensity after 24 h of the venom treatment.

Fig. 4B shows that no change was observed for the animals of PBS control group in which post- injection swelling completely disappeared in 12 h. On the other hand, the dermonecrotic lesion induced by venom reached its maximal size at 24 h of injection and steadily decreased over the period of 48 h. Topical application of tetracycline-mixed lanolin cream showed protective effect to some degree comparing with either lanolin cream alone or no topical treatment. Moreover, in the case of inoculation of tetracycline pre-mixed venom, it was capable of controlling the progression of the dermonecrotic lesion through the remainder of the experiment at all times. These results showed that NnV induces dermonecrotic lesions in rabbit skin and the pre-incubation of venom with tetracycline is most effective for preventing *N. nomurai* jellyfish venom-mediated dermonecrosis. Although it was less effective than tetracycline pre-incubation, the topical treatment with tetracycline cream presented the possibility of its use as a first aid for stinging by jellyfish species, such as *N. nomurai*.

### Histological Assessment of Skin Damage

**Figure 5:**
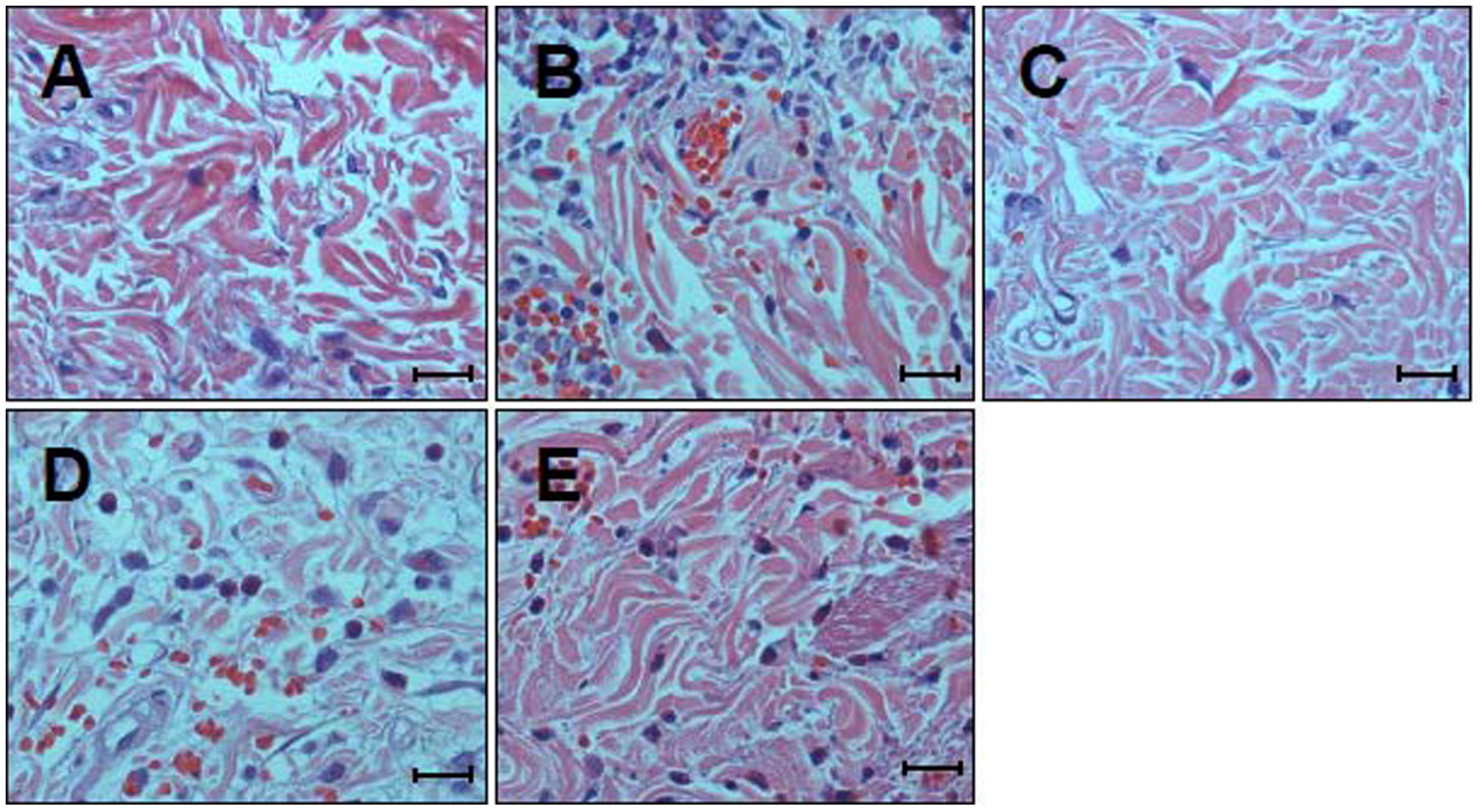
Histopathological examination of the dermonecrotic lesion induced by *N. nomurai* jellyfish venom in the absence or presence of tetracycline. New Zealand white rabbits were injected intradermally with PBS or *N. nomurai* jellyfish venom (500 µg) in the presence or absence of various treatments as described in Materials and methods. Each panel corresponds to the skin section injected with PBS (A); venom (B); tetracycline-pretreated venom (C); or venom, followed by the post-treatment with lanolin cream alone (D), or tetracycline cream (E), respectively. Representative sections are shown here and the similar results were obtained from all the rabbits examined (H&E; scale bar = 50 µm).

Histological sections of skin from PBS-injected group showed no observable changes and served as a negative control (Fig. 5A). Comparing with the control group, the skins from NnV-injected area showed various pathological changes, including dissociation of collagenous fiber, degeneration of muscle fibers owing to edema, moderate hemorrhage and intense neutrophil infiltration in the dermis (Fig. 5B). Histological analysis of skin sections injected with tetracycline pre-mixed NnV showed only minimal reaction characterized by low level of hemorrhage and neutrophil infiltration similar to that observed in PBS control group (Fig. 5C). This result suggests that dermal toxicity of *N. nomurai* jellyfish venom can be almost completely blocked by the incubation with tetracycline. On the other hand, the topical post-treatment of NnV-injected skin with lanolin cream showed some degree of dermonecrotic lesion profile that is similar to but a little less than those of NnV-injected skin (Fig. 5D). Whereas the post-treatment with tetracycline-added lanolin cream, though not complete, significantly reduced the hemorrhage as well as neutrophil infiltration induced by NnV injection. Moreover, it prohibited dissociation of collagenous fiber and degeneration of muscle fibers. These microscopic observations of the injection sites were consistent in all the tested rabbits.

## Discussion

There has been an increase in the reports of jellyfish envenomation in various coastal areas and it is estimated that in excess of 10,000 jellyfish stings occur each year around the world. Up to now at least 63 individuals had met sudden and painful deaths in Australia waters [20] and 14 deaths in Asian Pacific oceans [21] by the stings of poisonous jellyfish species. Not all species of jellyfish are poisonous even though they all have stinging cells, and the geographical distribution of venomous ones can be varied depending on the species. In the case of severe stings, however, the victims may show diverse responses, including fever, respiratory distress, and gastrointestinal symptoms of increased secretions, nausea, emesis, abdominal colic, and diarrhea [22], [23]. Major life threatening symptoms are cardiovascular instability with syncope, respiratory insufficiency, and anaphylaxis, which may result in the death of victims [21], [24], [25]. Besides, Jellyfish envenoming can also produce immediately painful tentacle-print dermatitis with pruritis and urticaria [26], [27] and/or delayed cutaneous reactions, characterized by eczematous lesions [28]. Taking into account the consequences of jellyfish envenomation on humans, there have been numerous investigations with the aim of preventing or reducing pathological effects derived from jellyfish venoms.

As an immediate emergency treatment, the use of antivenom, chemical, or enzyme inhibitor in the field has been proposed before hospital treatment for the stings and bites from various poisonous animals, such as snakes and jellyfish species [20], [29]. In the case of venom-induced skin damage, such intervention would reduce the action of venom before developing significant dermatological symptoms. Up to now, a number of chemical inhibitors (ethanol, meat tenderizer, ammonia, and vinegar) have been recommended for the treatment of jellyfish stings with variable efficacy depending on the reagent used as well as the jellyfish species examined. Among these, ethanol and acetic acid provided little or no relief from the pain and stinging sensation of jellyfish envenomation [30]. Recently, the development of MMP inhibitors, which has been tested in the clinical applications for a number of diseases, allows for the assessment of such inhibitors against the poisoning by venomous animals [31]. Tetracycline and its chemically modified ones are well-known MMP inhibitors and whose activity is based, among other suggested mechanisms, on the chelation of Zn^2+^- and Ca^2+^-ions, which are present in catalytic domain and needed for enzymatic activity [15] and/or the direct interaction with MMP proteins thereby inhibiting the enzyme [32]. Thus, tetracycline is of potential value for treating patients poisoned by venomous animals which have metalloproteinase-like enzymes in their venoms.

The gelatin zymography assay used in this study allowed the evaluation of a role of MMP-2 and MMP-9 induced by NnV in the local pathological alterations. NnV treatment induced an enhanced expression/secretion of MMP-2 and MMP-9 in HaCaT and NIH3T3 cells (Fig. 3 and 4). The activation of pro-MMP-2 is generally believed to require a proteolytic processing by MT1-MMP (MMP14). However, it is not yet clear the activation of pro-MMP-2 observed by the treatment of jellyfish venom is stimulated whether by MT1-MMP activity or the jellyfish venom metalloprotease(s). The issue will be further examined in the near future. The augmentation of MMPs expression/secretion turned out to be inversely proportional to the cell viability (Fig. 2). Moreover, both the cytotoxicity and MMPs expression/secretion can be almost completely suppressed by the use of tetracycline (Fig. 2, 3). The exposure of jellyfish venom to heat (37°C for 1 h) used during the preincubation of jellyfish venom with testing compounds has been proved to have negligible effect on the venom cytotoxic effect [4]. These data suggest that the beneficial effect of tetracycline involves the reduction of expression/secretion of MMPs and consequently the decrease in cell death.

Being consistent with the *in vitro* results, analysis of the action of tetracycline on an *in vivo* model suggested that jellyfish venom-mediated pathological changes in the affected skin tissue were significantly reduced or abrogated by the pre-incubation of NnV with tetracycline (Fig. 4, 5). These results positively correlate well with reduction of the lesion sizes in the animals (Fig. 4B), indicating that the spreading of the dermonecrotic lesion may be in part owing to the production of MMPs in the NnV-inoculated skin area. Likewise, other previous studies had also suggested that pre-incubation with inhibitor can be effective in preventing the toxic effects of box jellyfish venom in a cell-based assay [33] and animals [34]–[36]. On the other hand, treatment with topical application of lanolin cream and tetracycline cream were not able to fully block the local tissue damage process, which might already initiate before the start of the treatment (Fig. 5). This is also probably due to the delayed onset of action of tetracycline for the absorption and preventing rapid effects of the venom inoculated into local dermal tissues. It can be even more problematic when you consider the time delay that is likely to occur between a swimmer and fisherman being envenomed by *N. nomurai* jellyfish and receiving tetracycline. Nevertheless, the site of venom injection with topical application of tetracycline cream showed lower levels of hemorrhage and neutrophil infiltrations in the dermis comparing with those of venom alone or the treatment of lanolin cream (Fig. 5C). Therefore, though it has a limited clinical value, the formulation of cream type tetracycline for the topical use at the site of jellyfish sting still has some beneficial effects by protecting the venom-induced local tissue damage. This result suggests that tetracycline reagents may be used as a first-aid treatment for the affected skin area of jellyfish sting.

In conclusion, we have first demonstrated that *N. nomurai* jellyfish venom induces dermonecrotic effect by using both *in vitro* and *in vivo* models, and it involves an augmented expression and secretion of MMP-2 and MMP-9 by the venom. The finding of the ability of metalloproteinase inhibitor drugs, such as tetracycline, to reduce the necrotic dermal toxicity of *N. nomurai* jellyfish venom is novel and of potential therapeutic importance for other jellyfish venom stings as well. Although it may require further clinical study in the near future, our results imply that tetracycline may be useful for the therapy of dermonecrosis induced by jellyfish stings as a new therapeutic strategy. It is believed that there has been no previous report on the therapeutic agent of synthetic chemical origin for the treatment of jellyfish venom-induced dermonecrosis based on understanding its mechanism of action except the use of antivenom treatment. Furthermore, the current study, for the first time, has proposed a novel mechanism-based therapeutic intervention for skin damages caused by jellyfish stings.
